# Анализ содержания мелатонина и его взаимосвязь с дисфункцией яичников у пациенток репродуктивного возраста с ожирением (обзор литературы)

**DOI:** 10.14341/probl12710

**Published:** 2021-01-22

**Authors:** Р. К. Михеев, Е. Н. Андреева, Е. В. Шереметьева, Ю. С. Абсатарова, Т. А. Пономарева, О. Р. Григорян

**Affiliations:** Национальный медицинский исследовательский центр эндокринологии; Национальный медицинский исследовательский центр эндокринологии; Московский государственный медико-стоматологический университет им. А.И. Евдокимова; Национальный медицинский исследовательский центр эндокринологии; Национальный медицинский исследовательский центр эндокринологии; Национальный медицинский исследовательский центр эндокринологии; Национальный медицинский исследовательский центр эндокринологии

**Keywords:** мелатонин, ожирение: репродукция, инсулинорезистентность

## Abstract

Одним из новых направлений в изучении нарушений репродуктивной функции у женщин с ожирением являются влияние и рецепторная чувствительность мелатонина на гонадотропную функцию гипофиза и овариогенез с учетом хронологии «светового загрязнения». На современном этапе литературы по влиянию аспектов «светового загрязнения» на проблему ожирения и нарушений репродуктивной функции в отечественной и зарубежной литературе крайне мало. Данный обзор является попыткой объединить вышеуказанную проблему в рамках аспектов влияния «светового загрязнения» и уровня рецепторной чувствительности мелатонина у женщин репродуктивного возраста с ожирением. Поиск литературы проводили в отечественных (eLibrary, CyberLeninka.ru) и международных (PubMed, Cochrane Library) базах данных на русском и английском языках. Приоритетным являлся свободный доступ к полному тексту статей. Выбор источников был приоритетен периодом с 2015 по 2019 гг. Однако с учетом недостаточной изученности выбранной темы выбор источников датировался с 1992 г. Работа выполнена в рамках Государственного задания «Центральные и периферические патофизиологические механизмы развития болезней жировой ткани с учетом клинических и гормональных характеристик», 2020–2022 гг.

## Abstract (en)

One of the new directions in the study of reproductive disorders in obese women is the effect and receptor sensitivity of  melatonin on the gonadotropic function of the pituitary gland and ovariogenesis, taking into account the chronology of «light pollution». At the present stage, there is very little literature on the influence of the aspects of «light pollution» on the problem of obesity and reproductive disorders in the literature. This review is an attempt to combine the above problem in terms of the impact of «light pollution» and the level of receptor sensitivity of melatonin in women of reproductive age with obesity. The literature search was carried out in Russian (eLibrary, CyberLeninka.ru) and international (PubMed, Cochrane Library) databases in Russian and English. Free access to the full text of the articles was a priority. The selection of sources was prioritized for the period from 2015 to 2019. However, given the insufficient knowledge of the chosen topic, the choice of sources was dated from 1992. The work was carried out as part of the study «Central and peripheral pathophysiological mechanisms of development of adipose tissue diseases, taking into account clinical and hormonal characteristics» 2020–2022.

## ВВЕДЕНИЕ

В последние годы одним из новых и современных направлений в изучении нарушений репродуктивной функции у женщин с ожирением является проблема влияния мелатонина на гонадотропную функцию гипофиза/овариогенез; а также проблема хронологии «светового загрязнения». Однако на современном этапе данная проблема, являясь «пионерской», начала изучаться лишь у пациенток с синдромом поликистозных яичников (СПЯ), эндометриозом и у больных с онкологическими заболеваниями. При этом, по последним данным Всемирной организации здравоохранения (ВОЗ), в период с 1980 по 2013 гг. в мире отмечено увеличение доли взрослых, имеющих индекс массы тела (ИМТ) выше 25, с 28,8 до 36,9% у мужчин и с 29,8 до 38% у женщин. Согласно определению ВОЗ, ожирение — хроническое заболевание, вызываемое генетическими, метаболическими, поведенческими факторами, а также факторами окружающей среды, связанное с повышением частоты осложнений и смертностью [1]. Четкая тенденция к увеличению развития абдоминального ожирения у женщин с возрастом позволяет относить их в группу высокого риска развития обусловленных ожирением заболеваний. Увеличение абдоминальных жировых депо у женщин с андроидным типом ожирения связано с более выраженными изменениями показателей углеводно-жирового обмена. В настоящее время литературы по влиянию аспектов «светового загрязнения» на проблему ожирения и нарушений репродуктивной функции в отечественной и зарубежной литературе крайне мало. Данный обзор литературы является попыткой объединить проблему нарушений репродуктивной функции у женщин с ожирением в рамках аспектов влияния «светового загрязнения» и уровня мелатонина.

## МЕЛАТОНИН

Все биологические ритмы находятся в строгой подчиненности основному водителю ритмов, расположенному в супрахиазматических ядрах гипоталамуса. Гормоном-посредником, доносящим руководящие сигналы до органов и тканей, собственно и является мелатонин. При этом характер ответа регулируется не только уровнем гормона в крови, но и продолжительностью его ночной секреции. Кроме этого, мелатонин обеспечивает адаптацию эндогенных биоритмов к постоянно меняющимся условиям внешней среды. Регулирующая роль мелатонина универсальна для всех живых организмов, о чем свидетельствует присутствие этого гормона и четкая ритмичность его продукции у всех известных организмов, начиная с одноклеточных [2]. В рамках суточного ритма организма мелатонин поддерживает цикл сна/бодрствования, суточные изменения двигательной активности и температуры тела. Концентрация его в крови нарастает с наступлением темноты и достигает своего максимума за 1–2 ч до пробуждения. Изобретение более ста лет назад электричества и искусственного освещения кардинально изменило как световой режим, так и продолжительность воздействия света на человека. Воздействие света в ночное время, часто называемое «световым загрязнением», увеличилось и стало существенной частью современного образа жизни, что сопровождается множеством серьезных расстройств поведения и состояния здоровья. Согласно гипотезе «циркадианной деструкции», воздействие света в ночные часы нарушает эндогенный суточный ритм, подавляет ночную секрецию мелатонина, что приводит к снижению его концентрации в крови [2].

Тщательно проведенные исследования показали, что освещенность в 1,3 лк монохромного синего света или в 100 лк белого света может значительно подавить продукцию мелатонина эпифизом. Однако проблема «циркадной деструкции» на современном этапе оказалась более глобальной. Оказалось, что нарушение циркадной выработки мелатонина, рецепторной чувствительности к его воздействию замыкают «порочный круг» при многих онкологических, гинекологических заболеваниях, а также эндокринопатиях. При этом женщины являются более чувствительными к подавляющим эффектам ночного света на продукцию мелатонина, чем мужчины [2]. И более высокая предрасположенность женщин к эндокринопатиям, а именно — ожирению также способствует снижению выработки/нарушению рецепторной чувствительности к данному гормону.

На современном этапе проблема воздействия «световой загрязненности» на стероидогенез с научной позиции изучается лишь на животных; исследования на людях носят пилотный характер. При этом известно, что искусственное увеличение продолжительности светового периода в течение дня (на 2–4 ч) у грызунов приводит к увеличению продолжительности эстрального цикла и в некоторых случаях к его нарушению. Если воздействие света увеличить до 24 ч в сутки, у большинства мышей и крыс в короткие сроки развивается синдром персистирующего эструса. В физиологических условиях этот синдром развивается в более позднем возрасте (у крыс — обычно между 15 и 18 мес жизни) и затем переходит в анэструс, который является физиологическим эквивалентом периода менопаузы у женщин. При данном воздействии в яичниках у крыс с персистирующим эструсом обнаруживают фолликулярные кисты и гиперплазию тека-ткани яичника, также в них отсутствуют желтые тела. В противовес циклической продукции гонадотропинов, пролактина, эстрогенов и прогестерона, характеризующих физиологический репродуктивный период, эти гормоны секретируются ациклически, что приводит к гиперпластическим процессам в молочных железах и матке, повышая риск развития онкологических заболеваний [2]. Постоянное освещение приводит к увеличению порога чувствительности гипоталамуса к ингибирующему действию эстрогенов у самок крыс. Этот механизм является ключевым в старении репродуктивной системы как у самок крыс, так и у женщин. Таким образом, влияние света ночью приводит к ановуляции и ускорению связанного с возрастом выключения репродуктивной функции у грызунов и к дисменорее у женщин. Воздействие постоянного света увеличивает перекисное окисление липидов (ПОЛ) в тканях животных и уменьшает общую антиокислительную и супероксиддисмутазную активности, тогда как применение мелатонина вызывает снижение активности ПОЛ, особенно в головном мозге [2][3].

Однако если рассматривать проблему о влиянии мелатонина только с позиции «световой загрязненности», то данные клинических исследований о взаимосвязи работы в ночную смену и нарушении менструальной функции разноречивы. В исследовании здоровья медсестер (Nurses’ Health Study 3) приняли участие 6309 медсестер и студентов-медсестер США и Канады в возрасте от 21 до 45 лет. Из них 1196 медсестер (19%) сообщили о нерегулярных менструальных циклах, при этом 92 (1,5%), 935 (15,6%) и 789 (13,2%) участниц сообщили о продолжительности менструального цикла: <21 (очень короткий), 21–25 (умеренно короткий) и 32–50 (длинный) дней соответственно; нерегулярные менструальные циклы были более распространены среди женщин, работавших только ночью или со сменным графиком работы, по сравнению с женщинами, работавшими только в дневную смену, и были связаны с количеством ночных смен в месяц [3]. В противоположность этим данным исследование среди 766 медсестер в Норвегии не выявило никакой связи между ночной работой и нарушениями менструального цикла, а также не было выявлено различий в продолжительности и характере менструальных циклов среди медсестер, работавших по сменному графику [3].

## СИНДРОМ ПОЛИКИСТОЗНЫХ ЯИЧНИКОВ И МЕЛАТОНИН

До сих пор исследований по проблемам «световой загрязненности», «нарушения сна/бодрствования», а также суточной выработки мелатонина у пациенток c СПЯ в большой выборке не проводилось [4] (табл.  1). Известны лишь незначительные попытки устранить пробелы в данном вопросе. Так, в двух исследованиях проводилось сравнение уровней мелатонина в образцах крови/мочи между пациентками с СПЯ и контрольной группой. Результаты показали повышение уровня секреции с мочой 6-сульфатоксимелатонина (первичного метаболита мелатонина) у женщин с СПЯ (n=35 в первом исследовании, n=26 во втором исследовании) по сравнению с контрольной группой (n=35, n=26) [4][5]. Однако поскольку концентрация 6-сульфатоксимелатонина не показала явной корреляции с длительностью ночного сна, была случайно обнаружена другая корреляционная связь — между гиперандрогенией и концентрацией 6-сульфатоксимелатонина у данной категории больных [5]. А уровень мелатонина в плазме крови достоверно снижался параллельно снижению уровня общего тестостерона на фоне терапии комбинированным оральным контрацептивом с выраженным антиандрогенным эффектом (ципротерона ацетат + этинилэстрадиол) [5, 6]. Такая динамика изменений лабораторных показателей позволяет предположить наличие определенной патогенетической связи между секрецией мелатонина и выработкой андрогенов у пациенток с СПЯ. Однако данное предположение опровергает другое клиническое исследование. В процессе лечения СПЯ у пациенток, которым проводилась терапия мелатонином (2 мг/день в течение 6 мес), наблюдалось достоверное снижение уровня общего тестостерона и восстанавливалась менструальная функция, что, в свою очередь, позволяет предположить обратную связь между уровнями мелатонина и уровнями андрогенов в плазме крови [3][5].

**Table:** Таблица 1. Сравнительная характеристика исследований по изучению взаимосвязи между нарушениями сна и синдромом поликистозных яичников у пациенток репродуктивного периода [7]

Исследование, страна	Тип исследования	Группы исследования	Результаты
Hung et al.,Тайвань, 2006	Ретроспективное когортное исследование выборки, сформированное на основании базы данных Национального здравоохранения	5431 пациентка с СПЯ в анамнезе и 21 724 пациентки без СПЯ в анамнезе одной возрастной группы	Нарушения сна чаще диагностировались у женщин с СПЯ: коэффициент риска (HR) = 1,495 (95% ДИ 1,176–1,899). Нарушения сна наиболее часто манифестировали в 1-й год развития СПЯ
Lin et al., Тайвань, 2014	-	4595 пациенток с СПЯ и 4595 — без СПЯ одной возрастной группы	У женщин с СПЯ выявлялась более высокая частота развития нарушений сна (1,71 vs 0,63 на 1000 человек-лет). Коэффициент риска (HR) = 2,63 (95% ДИ 1,57–4,04), установленный в зависимости от демографических показателей (урбанизация, рождаемость) и коморбидных состояний (артериальная гипертензия, дислипидемия, сахарный диабет, ожирение)
Moran et al., Австралия, 2019	Перекрестное исследование когорты пациентов 1973–1975 гг. рождения из базы данных родильного дома	87 пациенток с СПЯ в анамнезе и 637 без СПЯ	Частота развития нарушений сна в 2 раза чаще у пациенток с СПЯ. Сложность засыпания: отношение шансов (OR) = 1,94 (95% ДИ 1,28–2,95). Сложность поддержки сна: отношение шансов (OR) = 1,92 (95% ДИ 1,12–3,31). СПЯ не взаимосвязан с ранним пробуждением или избыточной дневной сонливостью

Примечания: ДИ — доверительный интервал; HR — коэффициент риска; OR — отношение шансов.

СПЯ — комплексное эндокринное нарушение, затрагивающее такие аспекты женского здоровья, как обмен веществ, репродуктивная функция и психологическое равновесие женщины. Клиническими исследованиями было доказано, что частота встречаемости таких нарушений сна, как обструктивное апноэ сна и нарколепсия, у пациенток с СПЯ сравнительно выше, чем у пациенток контрольной группы. Интересен тот факт, что вышеперечисленные расстройства сна не только прямо коррелировали с ИМТ, но и встречались у пациенток с нормальной массой тела [7].

На сегодняшний день известны 2 этиопатогенетических механизма, демонстрирующих связь между СПЯ и нарушениями сна: гипоталамо-гипофизарный и психофизиологический. В основе первого механизма лежит гиперандрогения, приводящая к инсулинорезистентности, повышению уровню секреции кортизола и мелатонина; обсуждается также вопрос воздействия повышенного уровня контринсулярных гормонов и мелатонина на гипоталамо-гипофизарно-надпочечниковую ось. В основе второго механизма лежат факторы хронического стресса (тревога, депрессия), влекущие за собой негативные поведенческие реакции, выражающиеся в виде вредных привычек (курение, алкоголь, гиподинамия). Несмотря на то что многие звенья патогенеза нарушения сна у женщин с СПЯ до сих пор обсуждаются патофизиологами, последствия такого рода сложных взаимосвязей в долгосрочной перспективе являются предикторами повышения сердечно-сосудистого риска и риска развития сахарного диабета 2 типа. Именно поэтому диагностика и лечение нарушений сна у пациенток с СПЯ представляют собой междисциплинарную проблему, решение которой требует структурного подхода [7].

СПЯ — это патологическое состояние, при котором нарушается равновесие половых гормонов, что ведет к появлению тонкостенных образований в ткани яичников — кист. СПЯ всегда сопровождается нарушениями менструального цикла, бесплодием, поражением сердечно-сосудистой системы и негативными косметическими изменениями. Для создания новой теоретической модели ведения таких пациенток делаются попытки проведения исследований по изучению генетического полиморфизма мелатониновых рецепторов 1А и 1В у пациенток с ожирением и СПЯ. Так, пациентки с СПЯ, принявшие участие в одном из таких исследований, были разделены на 2 группы: пациентки с повышенным ИМТ (ИМТ≥25,5 кг/м²) и нормальным ИМТ; в контрольную группу вошли 215 пациенток с непроходимостью маточных труб и сохраненной овуляторной функцией. В ходе исследования контролировались такие показатели, как антропометрические данные, уровни половых гормонов, уровни липидов, глюкозы и инсулина в венозной плазме крови. Определение генотипа rs2119882 гена рецептора MTNR1A и генотипа rs10830963 рецептора MTNR1B проводилось молекулярно-генетическим методом (полимеразная цепная реакция (ПЦР) с определением полиморфизма длины рестрикционных фрагментов). Полученные результаты генного полиморфизма сравнивались между пациентами контрольной и экспериментальной групп. У пациенток контрольной группы с генотипом rs2119882, являвшихся носителем аллеля С, определялся повышенный риск развития СПЯ на фоне повышенного ИМТ. Носители TT-аллеля генотипа rs2119882 и СС-аллеля генотипа rs10830963 имели сниженный риск развития ожирения и повышенные риски развития гиперандрогении, гипоэстрогении и нарушений менструального цикла [8]. Таким образом, данное исследование подтвердило гипотезу о многофакторности нарушений мелатонинового обмена у женщин с эндокринопатиями, внося определенный вклад в попытки понимания проблемы влияния нарушений экспрессии мелатонина на оварио/стероидогенез.

## ОЖИРЕНИЕ

Ожирение, как сопутствующее СПЯ состояние, обуславливается метаболическим стрессом, возникающим вследствие характерной для данного заболевания относительной гиперандрогении [9, 10]. Согласно одному из исследований, частота встречаемости ожирения у пациенток с СПЯ составляет 49% (95% ДИ 42–55%), из которых 54% имеют ожирение по абдоминальному типу (95% ДИ 43–62%) [11]. А распространенность ожирения у пациенток с установленным диагнозом СПЯ в 2 раза выше по сравнению с женщинами с другими эндокринопатиями (ожирение: относительный риск (ОР) 2,8; 95% ДИ 1,9–4,1; абдоминальное ожирение: ОР 1,7; 95% ДИ 1,3–2,3) [3][12]. Надо отметить, что полученные статистические данные нельзя распространять на всех пациенток с СПЯ, поскольку частота встречаемости ожирения была выше в клинических исследованиях, чем по данным исследований среди компактно проживающих групп лиц [12]. В качестве наглядного примера можно привести данные многолетнего исследования, проведенного в Австралии (n=9145), по результатам которого у 4478 женщин был диагностирован СПЯ. У данных пациенток были выявлены в среднем более высокий ИМТ (±2,5 кг/м²; 95% ДИ 1,9–3,1) и более высокий темп прироста массы тела за 10-летний период ((±2,6 кг/м², 95% ДИ 1,2–4,0) по сравнению с их сверстницами без СПЯ [13].

Было бы некорректно говорить о наличии строго определенного ИМТ, характерного для когорты пациенток с СПЯ. Так, величина ИМТ во многом зависит от следующих факторов: возраст, этническая принадлежность и, вероятно, степень выраженности гиперандрогении. При этом попытка классифицировать вышеперечисленные факторы по степени тяжести на сегодняшний день терпит фиаско. Причина кроется в отсутствии данных о соотношении между гормональной и психофизиологической теориями возникновения СПЯ [11]. Существуют предположения о нарушениях механизма передачи сигнала сытости в центральную нервную систему и проявлении постпрандиального голода у пациенток с СПЯ [14]. Причинами являются нарушение грелин-опосредованного механизма регуляции аппетита с подавлением постпрандиальной выработки холецистокинина, ГПП-1 (глюкагоноподобного пептида типа 1) и пептида YY; энергетических затрат организма (1659 кДж/день), повышенная калорийность пищи (42 кДж в 1 порции), что эквивалентно набору массы тела со скоростью 1,9 кг/год [14–16]. Дополнительную роль в патогенезе СПЯ играют психологические стрессовые факторы, влекущие за собой тревожно-депрессивные расстройства, нарушения пищевого поведения, дисморфофобию, снижение качества жизни [17].

Ожирение является одним из самых предрасполагающих факторов для развития обструктивного апноэ сна в результате изменения синтопии верхних дыхательных путей и органов грудной клетки [18]. Многолетние исследования пациентов как мужского, так и женского пола показали, что увеличение массы тела на 10% увеличивает риск нарушений сна в 6 (!) раз [19]. При ИМТ >40 кг/м² риск развития обструктивного апноэ сна увеличивается на 92% [20].

На современном этапе установлено наличие связи между ожирением и развитием не только обструктивного апноэ сна, но и другими ассоциированными состояниями: избыточной дневной сонливостью, повышенной утомляемостью [21][22]. В 1998 г. зарубежными исследователями было выдвинуто предположение о наличии 2 возможных патогенетических взаимосвязей между ожирением и нарушениями дыхательной функции, в основе которых лежит гиперцитокинемия [23–25]. Первый механизм подразумевает собой снижение длительности периода сна в результате повышенной активности гипоталамо-гипофизарно-надпочечниковой оси. При втором механизме длительность периода сна не затрагивается, т.к. происходит развитие инсулинорезистентности вследствие гипо-/норморегуляции гипоталамо-гипофизарно-надпочечниковой оси.

Риск развития обструктивного апноэ сна вследствие ожирения также имеет тенденцию к увеличению у пациенток с сопутствующим СПЯ, что наглядно демонстрируют некоторые клинические исследования. Однако на современном этапе данные, подтверждающие/опровергающие данную гипотезу, противоречивы. В первом исследовании женщины с высоким ИМТ и СПЯ (n=53) имели повышенный риск развития обструктивного апноэ сна и дневной сонливости, снижения длительности ночного сна [25]. Во втором исследовании с использованием Берлинского опросника принимали участие 2 когорты испытуемых: женщины, имеющие (n=44) и не имеющие в анамнезе (n=34) СПЯ; было показано, что ожирение являлось ярким предиктором развития обструктивного апноэ сна [26]. В третьем же исследовании с участием женщин с ожирением и СПЯ (n=23) взаимосвязи между степенью увеличения ИМТ и выраженностью обструктивного апноэ сна (по индексу апноэ AHI) выявлено не было [27].

## ИНСУЛИНОРЕЗИСТЕНТНОСТЬ И СИНДРОМ ОБСТРУКТИВНОГО АПНОЭ

Многочисленными исследованиями была продемонстрирована достоверная связь между инсулинорезистентностью и обструктивным апноэ сна [28–30]. По данным межгруппового исследования по изучению факторов риска развития избыточной дневной сонливости (n=1741), проведенного в США, наличие сахарного диабета у пациенток с СПЯ (уровень глюкозы натощак >126 мг/дл) строго и прямо коррелировало с развитием избыточной дневной сонливости [31]. В другом исследовании, проведенном среди небольшой когорты пациентов мужского пола с избыточной дневной сонливостью (n=22), у испытуемых были выявлены более высокие уровни инсулина в венозной плазме крови по сравнению с группой контроля. При этом ИМТ в основной группе соответствовал нормативным показателям [31]. Проспективное исследование во Франции, посвященное частоте встречаемости обструктивного апноэ сна среди французских пациентов обеих полов (n=3565), также доказало взаимосвязь между гиперинсулинемией натощак и развитием обструктивного апноэ сна, однако независимо от уровня ИМТ [32]. Для снижения риска развития обструктивного апноэ сна у пациенток с инсулинорезистентностью было предложено использование препаратов, повышающих чувствительность рецепторов в инсулинзависимых тканях. Опыт лечения пациенток с СПЯ, получавших в ходе терапии метформин, показал более низкие показатели частоты развития нарушений сна и избыточной сонливости днем [33][34]. В другом рандомизированном плацебо-контролируемом исследовании с участием пациенток с/без сахарного диабета 2 типа контрольная группа получала плацебо, а основная — пиоглитазон. У пациенток, получавших пиоглитазон, согласно показателям уровней инсулина в венозной плазме крови натощак, снизилась инсулинорезистентность, однако не произошло видимого улучшения со стороны обструктивного апноэ [34]. Решение данной клинической проблемы заключается в проведении значительно большего числа исследований с более обширной выборкой испытуемых и использованием современных методов диагностики как инсулинорезистентности (клэмп-метод как золотой стандарт), так и оценки синдрома обструктивного апноэ [34].

На сегодняшний день объяснить патогенетическую взаимосвязь между инсулинорезистентностью и развитием обструктивного апноэ сна, а также избыточной дневной сонливостью не представляется возможным. Однако экспериментально доказано, что в целом развитие данных патологий можно однозначно объяснить повышением активности симпатической нервной системы в результате гиперинсулинемии, вследствие чего укорачивается длительность периода сна [33][34]. А вот какова роль мелатонина и проблемы «световой загрязненности» в возникновении данных патологических процессов — задача, которую необходимо решать в ближайшее время.

## ЗАКЛЮЧЕНИЕ

В результате обзора и анализа ряда клинических исследований выявлено, что при наличии ожирения проблема нарушения циркадности уровня мелатонина, а также его рецепторной чувствительности явно подтверждается взаимодействием нарушения работы гипоталамо-гипофизарно-надпочечниковой системы, метаболического стресса и нарушения архитектоники сна. По данной причине мы имеем полное право предполагать, что решение данной проблемы является мультидисциплинарной задачей с необходимостью привлечения консилиума различных специалистов (гинеколога, эндокринолога, сомнолога, психиатра, диетолога, врача ЛФК и т.д.). Однако на современном этапе имеющиеся данные более характерны для уровней доказательности В и С, но при этом могут помочь нам в построении стратегии ведения пациенток репродуктивного возраста с ожирением/избыточной массой тела.
